# A Compound of Chinese Herbs Protects against Alcoholic Liver Fibrosis in Rats via the TGF-*β*1/Smad Signaling Pathway

**DOI:** 10.1155/2019/9121347

**Published:** 2019-04-22

**Authors:** Xiaomeng Li, Yunjie Liu, Wuyang Yue, Yuefeng Tan, He Wang, Lishi Zhang, Jinyao Chen

**Affiliations:** ^1^West China School of Public Health and West China fourth Hospital, Sichuan University, Chengdu, China; ^2^Food Safety Monitoring and Risk Assessment Key Laboratory of Sichuan Province, Chengdu, China

## Abstract

Alcoholic liver fibrosis (ALF) has become a major public health concern owing to its health impacts and the lack of effective treatment strategies for the disease. In this study, we investigated the effect of a compound composed of Chinese herbs* Pueraria lobata* (Willd.),* Salvia miltiorrhiza*,* Schisandra chinensis*, and* Silybum marianum* on ALF. An ALF model was established. Rats were fed with modified Lieber–Decarli alcohol liquid diet and injected with trace CCl_4_ at late stage. The rats were then treated with several doses of the compound. Biochemical and fibrosis-relevant parameters were measured from the sera obtained from the rats. Liver tissues were obtained for hematoxylin and eosin and Masson's trichrome staining. Matrix metalloproteinase-13 and tissue inhibitor of metalloproteinase-1 were determined by immunohistochemistry assays. The mRNA and protein expression levels of transforming growth factor-*β*1 (TGF-*β*1), Smad2, Smad3, and Smad7 on the livers were also measured by quantitative polymerase chain reaction and Western blot. Results showed that the compound treatment alleviated pathological lesions in the liver, decreased the serum levels of hyaluronan, laminin, and hydroxyproline, and diminished the expression of hepatic tissue inhibitor of metalloproteinase-1. Compound treatment also increased hepatic matrix metalloproteinase-13 expression and inhibited the TGF-*β*1/Smad signaling pathway. In conclusion, the compound has a protective effect against ALF in rats, and an underlying mechanism is involved in the TGF-*β*1/Smad signaling pathway.

## 1. Introduction

Alcoholic liver disease (ALD) is an important public health problem with wide pathologic spectrum, including steatosis, steatohepatitis, fibrosis, and cirrhosis [1]. These pathologic manifestations can exist simultaneously, successively, or independently [2]. Alcoholic liver fibrosis (ALF) is a reversible turning point and progresses into cirrhosis and even hepatocellular carcinoma if unsuccessfully treated. In recent decades, great efforts have been made to explore prospect therapeutic medicines for ALF, but effective strategies of treatment are still not available. Meanwhile, Chinese traditional herbal medicines have been demonstrated to be promising in preventing and even reversing the ALD progress (ALF included) [3].

Chinese traditional herbs* Pueraria lobata* (Willd.),* Salvia miltiorrhiza, Schisandra chinensis, *and* Silybum marianum* can ameliorate chemical-induced liver fibrosis [4–7] and alcohol-induced acute and chronic lesions in the liver [8–11]. However, the effects of these herbs on ALF are rarely reported. In traditional Chinese medical theory, herbs tend to be prescribed concomitantly to enhance efficacy and reduce the side effects [12, 13]. The combined protective effects of these herbs on ALF and potential mechanisms have not been investigated yet.

Liver fibrosis is characterized as an excessive accumulation of extracellular matrix (ECM) generated by activated hepatic stellate cells (HSCs) [14]. HSC activation, a key event in liver fibrosis, is a complicated process comprising two major stages: initiation and perpetuation [15]. The transforming growth factor-*β* (TGF-*β*) is a vital molecule that promotes the stimulation of collagen I and other ECMs produced by HSCs [16]. Signals downstream of TGF-*β* are known as Smads proteins.

In this study, we established an ALF rat model to investigate the possible protective effect of the compound and its underlying mechanism in order to provide evidence for the application of Chinese traditional herb therapies in ALF prevention and treatment.

## 2. Materials and Methods

### 2.1. Chemicals

The compound used in this study was provided by the By-Health Co., Ltd. (Zhuhai, China). The compound is a mixture of extracts from four kinds of Chinese herbs, namely,* P. lobata* (Willd.) (40%),* S. miltiorrhiza *(25%)*, S. chinensis *(20%), and* S. marianum *(15%) (abbreviated as PSSS compound). Ethanol, CCl_4_, and olive oil, all analytical grade, were purchased from Chengdu Kelong Chemical Reagent Factory (Chengdu, China).

### 2.2. Animals and Treatment

Male Sprague–Dawley rats, Specific Pathogen Free, weighing approximately 150±20 g, were obtained from Sichuan Academy of Chinese Medicine Sciences (Certificate No. SCYK 2013-011) (Chengdu, China). All animals were acclimatized to the laboratory environment for 1 week before the experimental procedures and housed under constant temperature, appropriate humidity, and a 12 h light/dark cycle. Animal treatment protocol followed the guidance of the Ethical Committee for Research on Laboratory Animals of Sichuan University.

Sixty-three rats were randomly divided into two groups: the blank control group (n=9) and ALF model group (n=54). ALF model animals received modified Lieber–Decarli alcohol liquid diet (energy source: 30% from alcohol, 35% from fat, 18% from protein, and 17% from carbohydrate; Trophic Animal Feed High-Tech, Nantong, China), plus 25% CCl_4_ (intraperitoneally; 0.4 mL/kg·bw/day, dissolved in olive oil) once a week from the ninth week of Lieber–Decarli diet feeding for five times. The blank control group intraperitoneally received nonalcoholic liquid diet (energy source: 35% from fat, 18% from protein, and 47% from carbohydrate) and equivalent volume of normal saline. Rats were all kept in single cage, and the feeding amount of all animals was adjusted to deliver equivalent calories.

**Figure 1:**
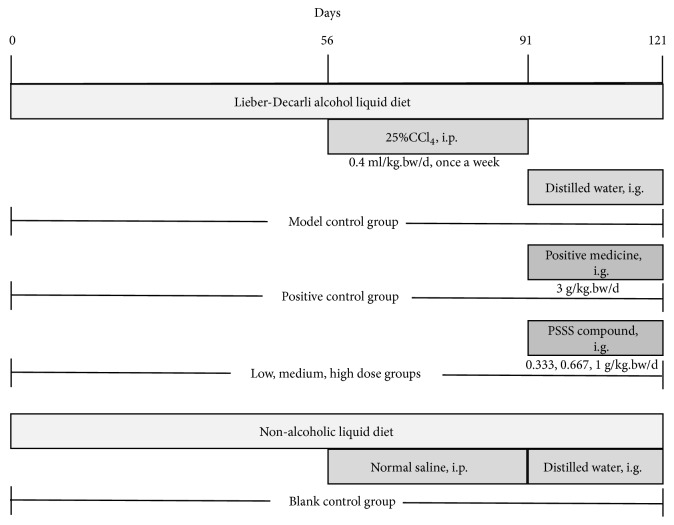
Experimental protocol on model establishment and test substance administration. Notes: i.p., intraperitoneally; i.g., intragastrically.

After successful establishment of the ALF model, 44 rats in the ALF model group (10 rats were sacrificed to determine establishment of the ALF model) were randomly divided into five groups. The model control group was composed of ALF rats gavaged with distilled water; the positive control group was composed of ALF rats that received liver fibrosis treatment medicine (Fufangbiejiaruanganpian; 3 g/kg·bw/day) approved by the China Food and Drug Administration; the low, medium, and high dose groups were composed of ALF rats that received 0.333, 0.667, and 1 g/kg·bw/day of PSSS compound, respectively; and the blank control group was composed of rats that received distilled water alone (Figure 1). All groups were treated by oral gavage once a day.

After treatment for 30 days, all rats were anesthetized with 7% chloral hydrate and then sacrificed. The liver, kidney, spleen, testis, and thymus samples were obtained and weighed. A fraction of each serum sample was used for the measurement of biochemical and fibrosis-relevant parameters, and the rest was kept frozen at −80°C until assayed. One piece of liver was fixed in paraformaldehyde (4%) solution for histological analysis, and the other was frozen at −80°C until used.

### 2.3. Measurement of Index of Organs

(1) $$\begin{array}{c} Index\;\;of\;\;organs=\left(\;\frac{Absolute\;\;organs\;\;weight}{Body\;\;weight}\;\right)\times 100. \end{array}$$

### 2.4. Histological Analysis

The same portion of right lobe of the liver was fixed in paraformaldehyde (4%) solution. Paraffin-embedded 5 *μ*m-liver sections were stained with hematoxylin and eosin (H&E) for histological evaluation and Masson's trichrome for fibrosis assessing. Images were captured with an Olympus BX 53 light microscope (Tokyo, Japan) and an Olympus DP73 CCD (Tokyo, Japan) at 200× magnification. Steatosis, hepatitis, and fibrosis were scored according to Chinese Guidelines for management of alcoholic liver disease: an updated and revised edition [17].

### 2.5. Biochemical Parameters in the Serum

The serum levels of alanine aminotransferase (ALT), aspartate aminotransferase (AST), alkaline phosphatase (ALP), *γ*-glutamyl transpeptidase (GGT), and triglyceride (TG) were measured by an Au 400 automatic biochemical analyzer (Olympus, Tokyo, Japan).

### 2.6. Determinations of Hyaluronan (HA), Laminin (LN), and Hydroxyproline (Hyp) in the Serum

HA, LN, and Hyp levels in the sera were assayed with commercially available enzyme-linked immunosorbent assay kits (Cusabio Biotech, Wuhan, China) following the manufacturer's instructions.

### 2.7. Immunohistochemistry Assays of Matrix Metalloproteinase-13 (MMP-13) and Tissue Inhibitors of Metalloproteinase-1 (TIMP-1) in the Liver

Immunohistochemistry assays were performed using rabbit anti-MMP-13 (dilution 1:200, Proteintech group, Wuhan, China) and anti-TIMP-1 (dilution 1:50, Proteintech group, Wuhan, China). Quantitative analysis was conducted with ImageJ software (version 1.8.0_112, National Institutes of Health, USA).

### 2.8. mRNA Expression Levels of TGF-*β*1, Smad2, Smad3, and Smad7 in the Liver

The mRNA expression levels of TGF-*β*1, Smad2, Smad3, and Smad7 were determined by real-time quantitative polymerase chain reaction (qPCR) analysis. Total RNA was extracted from frozen liver tissues by using a Trizol reagent (Tiangen Biotech, Beijing, China) and then reversely transcribed with a cDNA synthesis kit (Tiangen Biotech, Beijing, China) according to the manufacturer's instructions. Primer sequences were synthesized by Tsingke Biotech Co., Ltd. (Chengdu, China), and are listed in Table 1. qPCR was conducted using a Fast qPCR Mix kit (Tsingke Biotech, Chengdu, China) at 95°C for 1 min, followed by 40 cycles of 95°C for 10 s and 60°C for 10 s with BIO-RAD CFX96 real-time system. *β*-Actin was used as the endogenous reference gene, and target gene expression levels were analyzed by 2^−ΔΔCt^ method.

### 2.9. Protein Expression Levels of TGF-*β*1, Smad2, Smad3, and Smad7 in the Liver

Western blot was conducted to determine the protein expression levels of TGF-*β*1, Smad2, Smad3, and Smad7. Total protein was obtained from frozen liver tissues (100 mg) by homogenization with cold 1 ml lysis buffer. Then, the concentrations were measured by a bicinchoninic acid protein assay kit (Beyotime Biotech, Shanghai, China). Proteins were separated by 10% sodium dodecyl sulfate-polyacrylamide gel electrophoresis gel and transferred onto polyvinylidene fluoride (PVDF) membranes (Millipore, USA). PVDF membranes were blocked in 5% bovine serum albumin dissolved in tris-buffered saline containing Tween-20 (TBST) for 2 h and then incubated in primary rabbit anti-rat TGF-*β*1 (monoclonal, 1:1000, Abcam, USA), rabbit anti-rat p-Smad2, rabbit anti-rat Smad2, rabbit anti-rat p-Smad3, rabbit anti-rat Smad3 (all monoclonal, 1:1000, CST, USA), rabbit anti-rat Smad7 (polyclonal, 1:1000, Proteintech group, Wuhan, China), and rabbit anti-rat GADPH (polyclonal, 1:10000, Proteintech group, Wuhan, China) overnight at 4°C. After washing by TBST, the membranes were incubated with HRP-conjugated affinipure goat anti-rabbit secondary antibodies (1:1000, Proteintech group, Wuhan, China) for 1 h. Subsequently, proteins were visualized with enhanced chemiluminescence solution (Millipore, USA) and BIO-RAD ChemiDoc XRS+. The relative expression of proteins was analyzed using ImageJ software (version 1.8.0_112, National Institutes of Health, USA).

### 2.10. Statistical Analysis

After normality test and homogeneity test of variance, one-way ANOVA analysis followed by Tukey's test or Dunnett-t test (Tukey's test was used when samples of all groups were equal in size, if not, Dunnett-t test was used) for comparisons between groups was performed using SPSS 20.0 software (Chicago, USA).* P*<0.05 was considered statistically significant.

## 3. Results

### 3.1. Body Weight and Index of Organs

One rat in the model control group died because of liver injury. No statistical differences were observed in body weight and index of the liver, kidneys, spleen, testis, and thymus among the sacrificed groups (Table 2).

### 3.2. Histological Observations

**Figure 2:**
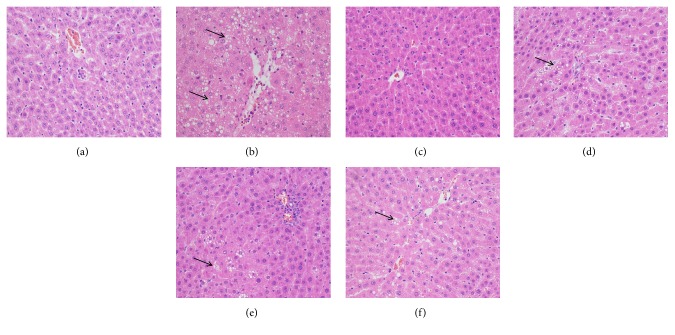
H&E staining of hepatic histological observations (magnification, ×200). Notes: arrows represent fat vacuoles. (a) Blank control group (distilled water). (b) Model control group (distilled water). (c) Positive control group (3 g/kg·bw/day, positive medicine). (d) Low dose group (0.333 g/kg·bw/day, PSSS compound). (e) Medium dose group (0.667 g/kg·bw/day, PSSS compound). (f) High dose group (1 g/kg·bw/day, PSSS compound).

**Figure 3:**
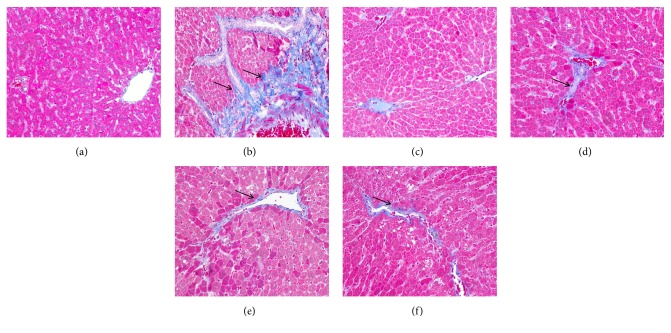
Masson' trichrome staining of hepatic histological observations (magnification, ×200). Notes: arrows represent collagen deposition. (a) Blank control group (distilled water). (b) Model control group (distilled water). (c) Positive control group (3 g/kg·bw/day, positive medicine). (d) Low dose group (0.333 g/kg·bw/day, PSSS compound). (e) Medium dose group (0.667 g/kg bw/day, PSSS compound). (f) High dose group (1 g/kg bw/day, PSSS compound).

In the H&E stains (Figure 2), the shapes of the liver cells were normal, nuclei were visible, and only a small amount of inflammatory cells and a slight level of steatosis can be observed in the portal area in the blank control group. In the model control group, normal structure of liver tissue was destroyed, hepatocytes were degenerated and necrotic, and macrobubble-like steatosis and disordered hepatic cord arrangement were observed. Compared with the model control group, the degrees of liver tissue destruction and steatosis were reduced in the positive control, low, medium, and high dose groups. Masson' trichrome staining (Figure 3) showed that in the blank control group, only minute collagen deposition can be observed in the portal area. In the model control group, a large amount of collagen deposition was observed in the pericellular and portal area. In the positive control, low, medium, and high dose groups, collagen deposition was apparently less than those of the model control group.

**Figure 4:**
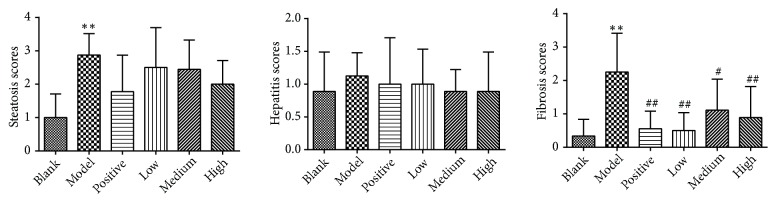
Scores of hepatic histological observations. Notes: data are shown as mean±SE (n=8 or 9). One-way ANOVA analysis followed by Dunnett-t test was conducted to calculate statistical significance. *∗∗P*<0.01 versus the blank control group; ^#^*P*<0.05 and ^##^*P*<0.01 versus the model control group.

As shown in Figure 4, compared with the blank control group, steatosis and fibrosis scores in the model control group significantly increased (*P*<0.01). Fibrosis scores in the positive control and PSSS compound groups significantly decreased compared with the model control group (*P*<0.05 or* P*<0.01). The histological results suggest that the ALF rat model was established successfully, and PSSS compound plays a protective role on ALF.

### 3.3. Serum Levels of Biochemical Parameters

**Figure 5:**
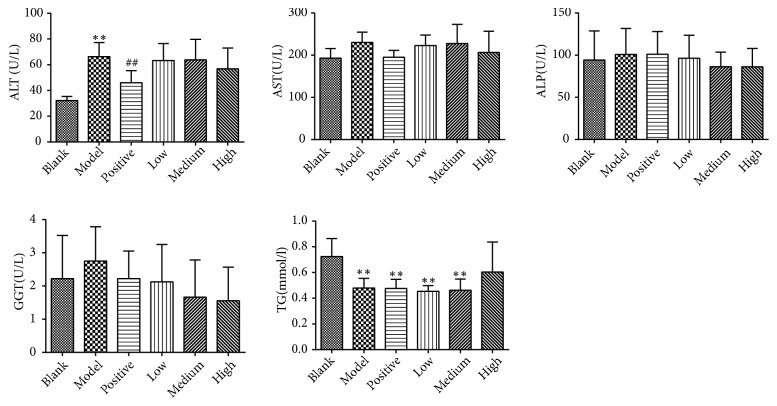
Serum levels of ALT, AST, ALP, GGT, and TG. Notes: data are shown as mean±SE (n=8 or 9). One-way ANOVA analysis followed by Dunnett-t test was conducted to calculate statistical significance. *∗∗P*<0.01 versus the blank control group; ^##^*P*<0.01 versus the model control group. ALT, alanine aminotransferase; AST, aspartate aminotransferase; ALP, alkaline phosphatase; GGT, *γ*-glutamyl transpeptidase; TG, triglyceride.

No statistically significant differences of serum AST, ALP, and GGT levels between groups were observed. Serum ALT levels in the model control group were significantly elevated compared with the blank control group, whereas the ALT levels in the positive control group were significantly lower compared with that in the model control group (*P*<0.01). Serum TG levels in the model control, positive control, and PSSS compound groups were all significantly lower than those in the blank control group (*P*<0.01) (Figure 5).

### 3.4. Serum Levels of HA, LN, and Hyp

**Figure 6:**
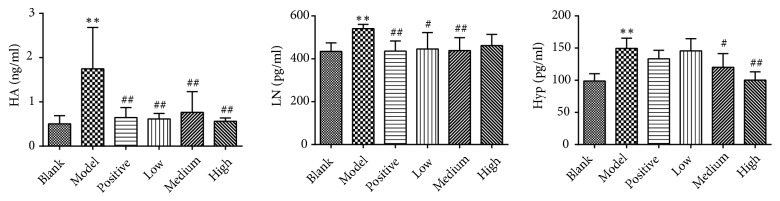
Serum levels of HA, LN, and Hyp. Notes: data are shown as mean±SE (n=6). One-way ANOVA analysis followed by Tukey's test was conducted to calculate statistical significance. *∗∗P*<0.01 versus the blank control group; ^#^*P*<0.05 and ^##^*P*<0.01 versus the model control group. HA, hyaluronan; LN, laminin; Hyp, hydroxyproline.

As shown in Figure 6, in the model control group, the serum levels of HA, LN, and Hyp, all vital indicators of liver fibrosis, were significantly increased compared with those in the blank control group (*P*<0.01). Compared with the model control group, the serum levels of HA were significantly decreased in the positive control and PSSS compound groups (*P*<0.01). The serum levels of LN were significantly decreased in the positive control, low, and medium dose groups (*P*<0.05 or* P*<0.01). The serum levels of Hyp were significantly decreased in the medium and high dose groups (*P*<0.05 or* P*<0.01). These results also demonstrate antifibrotic effect of the PSSS compound.

### 3.5. Expression Levels of Hepatic MMP-13 and TIMP-1

**Figure 7:**
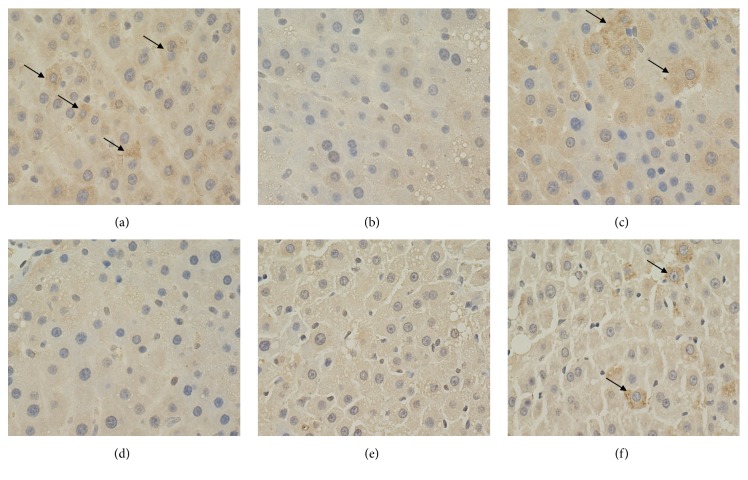
Expression levels of hepatic MMP-13 (magnification, ×400). Notes: arrows represent the positive expression of MMP-13. (a) Blank control group (distilled water). (b) Model control group (distilled water). (c) Positive control group (3 g/kg·bw/day, positive medicine). (d) Low dose group (0.333 g/kg·bw/day, PSSS compound). (e) Medium dose group (0.667 g/kg·bw/day, PSSS compound). (f) High dose group (1 g/kg·bw/day, PSSS compound). MMP-13, matrix metalloproteinase-13.

**Figure 8:**
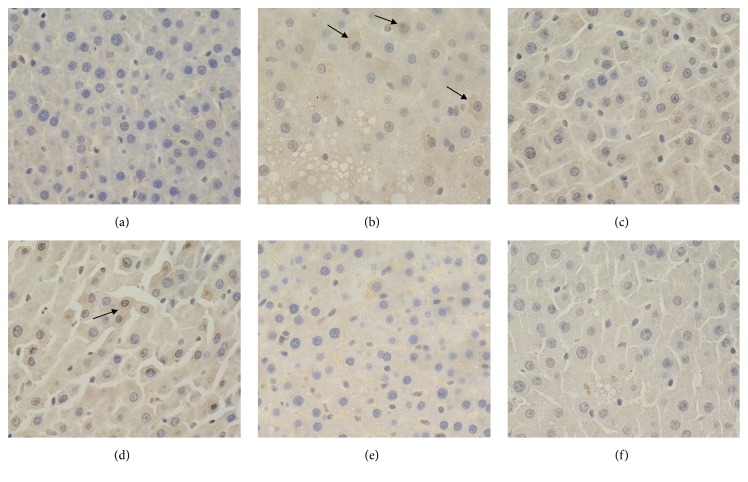
Expression levels of hepatic TIMP-1 (magnification, ×400). Notes: arrows represent the positive expression of TIMP-1. (a) Blank control group (distilled water). (b) Model control group (distilled water). (c) Positive control group (3 g/kg·bw/day, positive medicine). (d) Low dose group (0.333 g/kg·bw/day, PSSS compound). (e) Medium dose group (0.667 g/kg·bw/day, PSSS compound). (f) High dose group (1 g/kg·bw/day, PSSS compound). TIMP-1, tissue inhibitor of metalloproteinase-1.

**Figure 9:**
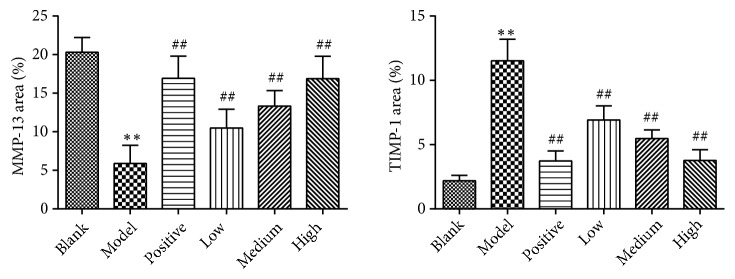
Quantitative analysis of the expression levels of hepatic MMP-13 and TIMP-1. Notes: data are shown as mean±SE (n=5). One-way ANOVA analysis followed by Tukey's test was conducted to calculate the statistical significance. *∗∗P*<0.01 versus the blank control group; ^##^*P*<0.01 versus the model control group. MMP-13, matrix metalloproteinase-13; TIMP-1, tissue inhibitor of metalloproteinase-1.

A significant reduction of hepatic MMP-13 and increase of hepatic TIMP-1 were observed in the model control group compared with those in the blank control group. Moreover, the changes were significantly inhibited by PSSS compound administration (*P*<0.01) (Figures 7, 8, and 9), suggesting protective effect of the compound involves MMP-13 and TIMP-1.

### 3.6. mRNA and Protein Expression Levels of Hepatic TGF-*β*1/Smad Signaling Pathway

**Figure 10:**
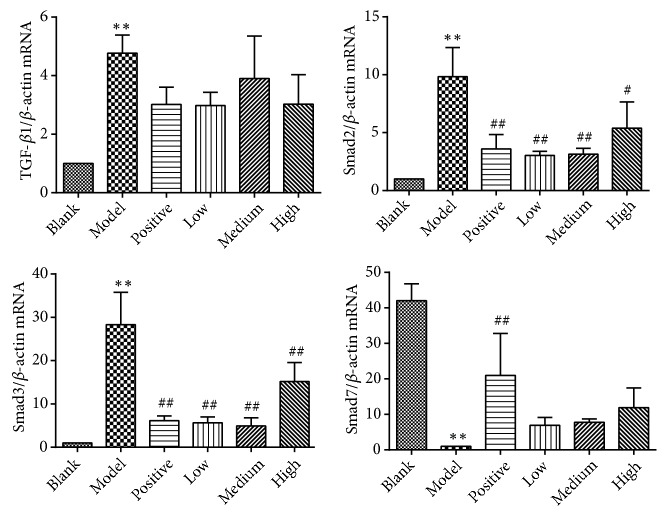
mRNA relative expression levels of hepatic TGF-*β*1, Smad2, Smad3, and Smad7. Notes: data are shown as mean±SE (n=3). One-way ANOVA analysis followed by Tukey's test was conducted to calculate statistical significance. *∗∗P*<0.01 versus the blank control group; ^#^*P*<0.05 and ^##^*P*<0.01 versus the model control group. TGF-*β*1, transforming growth factor-*β*1.

**Figure 11:**
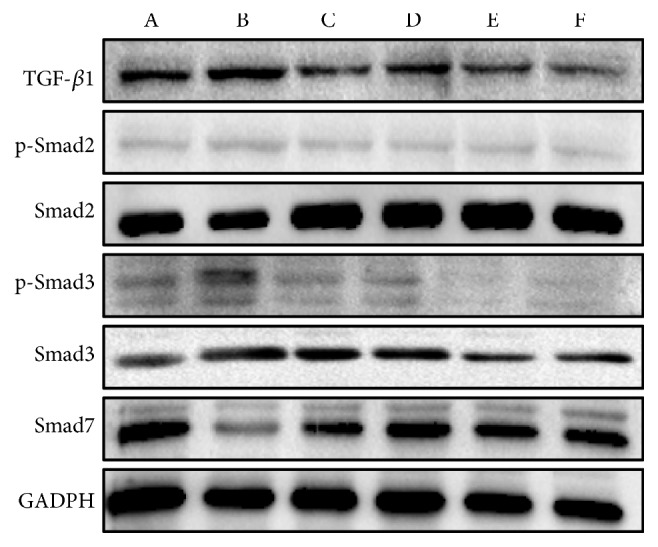
Protein expression levels of hepatic TGF-*β*1, p-Smad2, Smad2, p-Smad3, Smad3, and Smad7. Notes: A: blank control group (distilled water); B: model control group (distilled water); C: positive control group (3 g/kg·bw/day, positive medicine); D: low dose group (0.333 g/kg·bw/day, PSSS compound); E: medium dose group (0.667 g/kg·bw/day, PSSS compound); F: High dose group (1 g/kg·bw/day, PSSS compound). TGF-*β*1, transforming growth factor-*β*1.

**Figure 12:**
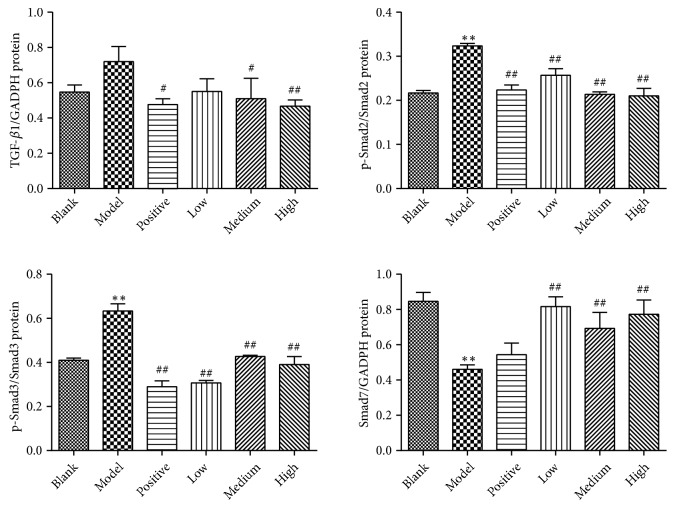
Semiquantitative analysis of the relative expression levels of hepatic TGF-*β*1, p-Smad2, p-Smad3, and Smad7. Data are shown as mean±SE (n=3). One-way ANOVA analysis followed by Tukey's test was conducted to calculate statistical significance. *∗∗P*<0.01 versus the blank control group; ^#^*P*<0.05 and ^##^*P*<0.01 versus the model control group. TGF-*β*1, transforming growth factor-*β*1.

As shown in Figures 10, 11, and 12, in the model control group, relative mRNA and/or protein expression levels of hepatic TGF-*β*1, (phospho-)Smad2, and (phospho-)Smad3 were significantly higher, and the Smad7 level was significantly lower than that of the blank control group (*P*<0.05 or* P*<0.01). Compared with the model control group, the protein expression levels of TGF-*β*1 were significantly lower in the positive control and the medium and high dose groups (*P*<0.05 or* P*<0.01). Both mRNA and protein expression levels of (phospho-)Smad2 and (phospho-)Smad3 were significantly lower in the positive control and PSSS compound groups (*P*<0.05 or* P*<0.01). The protein expression levels of Smad7 were significantly high in the PSSS compound groups (*P*<0.01). These results suggest that ALF alleviation of PSSS compound involves TGF-*β*1/Smad signaling pathway.

## 4. Discussion

ALF is an important health problem, causing tremendous disease burden and medical expenses. Thus, research on its pathogenesis and potential therapeutic agents are of great concern in medical science. The establishment of an ALF animal model is necessary to explore latent treatment remedy, and an ideal ALF animal model should have similar pathological characteristics to human ALF. The models of Lieber–Decarli alcohol liquid diet feeding ad libitum have been widely used [18], whereas applying alcohol alone is not enough to induce liver fibrosis in animals under laboratory environment [19]. Therefore, in this study, the ALF animal models were developed by a secondary agent (CCl_4_) accompanied with chronic alcohol treatment, called a “two-hit” model [20].

In traditional Lieber–Decarli diet, alcohol supplies calories of as high as 36%. However, rats' natural aversion to alcohol may affect the intake of this diet and cause nutritional deficiencies [21]. In this study, we successfully developed an ALF rat model by 13-week feeding of Lieber–Decarli alcohol liquid diet with reducing alcohol calories (30%), plus trace CCl_4_ injection in the subsequent 5 weeks. Pathological changes similar to human ALF, which includes steatosis, hepatocellular damage (ballooning), and a variable degree of pericellular and lobular fibrosis typically manifested in ALF [22], were replicated in our rat model (Figures 2 and 3).

In recent decades, a series of studies reported that extracts or a compound from herb exhibited protective effects on ALD [23–25], ALF included [20, 26], certainly.* P. lobata* (Willd.),* S. miltiorrhiza*,* S. chinensis*, and* S. marianum* have all been reported to have potential capability in alleviating ALD [25, 27–29] and chemical-induced liver fibrosis [6, 30–32]. However, to the best of our knowledge, no report on the effect on ALF of the combination of the four herbs is available. Interestingly, combination of traditional Chinese herbs has been practiced for thousands of years and can strengthen the efficacies. Therefore, in this study, four Chinese herbs were administered collectively, and the compound significantly decreased the liver fibrosis scores, indicators of liver fibrosis, i.e., serum levels of HA, LN, and Hyp, indicating beneficial role on ALF of this compound.

TGs are mainly synthesized, secreted, and catabolized in the liver [33]. As a result, TG synthesis would be disrupted when liver function is impaired, and serum levels of TG can be reduced. Our study found a significant decline in the serum levels of TG in the model control group, the positive control group, and the PSSS compound groups, probably due to damaged rat liver function or not fully recovered liver function in these groups.

ECMs are degraded by MMPs regulated by their inhibitors, i.e., TIMPs [34]. MMP-13 and TIMP-1 play a crucial role in modulation of liver fibrosis in rodents [35]. In the present study, a significantly decreased expression of hepatic TIMP-1 and an increased expression of hepatic MMP-13 in the PSSS compound groups demonstrate that the antifibrotic effect relates to regulation of ECMs via MMP-13 and TIMP-1.

TGF-*β*1 plays an essential role in fibrotic diseases, which can directly or indirectly bind to TGF-*β* type II receptors, and then phosphorylate and activate TGF-*β* type I receptors [36]. Activated type I TGF-*β* receptors phosphorylate Smad2 and Smad3, which bind to Smad4 and form a complex [37]. The complex then translocates from the cytoplasm into the nucleus and interacts with other transcription factors [38]. In addition, Smad6 or Smad7 is an inhibitive factor in the phosphorylation of Smad2 and Smad3 [39]. In our study, the compound treatment significantly decreased relative mRNA and/or protein expression levels of hepatic TGF-*β*1, (phospho-)Smad2, and (phospho-)Smad3 but increased the Smad7 expression. The changes of TGF-*β*1 and Smads are consistent with previous studies about the molecular mechanism of liver fibrosis [39–41], suggesting that the protective effect of the compound is via TGF-*β*1/Smad signaling pathway.

It is still not fully clear about which categories of bioactive components could effectively reverse alcoholic liver fibrosis. Puerarin, salvianolic acid A, schisandrin B, and silymarin, major bioactive components of* P. lobata* (Willd.),* S. miltiorrhiza*,* S. chinensis,* and* S. marianum*, respectively, have been reported to can alleviate CCl4-induced liver fibrosis or chronic alcoholic liver injuries [6, 7, 9, 31]. Furthermore, compound used in our study is rich in these bioactive components. Thus, it is reasonable to presume that these bioactive components may be the functional components for ALF. Nevertheless, identifying and maybe extracting bioactive components using the instrumental analysis, from these Chinese herbs, i.e.,* P. lobata *(Willd.)*, S. miltiorrhiza, S. chinensis, and S. marianum*, are surely a direction of our future study.

## 5. Conclusion

This study demonstrates that a compound of four Chinese herbs, namely,* P. lobata* (Willd.),* S. miltiorrhiza, S. chinensis*, and* S. marianum*, can be a novel therapeutic agent on preventing and reversing ALF, and the underlying mechanism of the protective effects may involve TGF-*β*1/Smad signaling pathway.

## Figures and Tables

**Table 1:** qPCR primer sequences used in this study.

Genes	Forward 5'-3'	Reverse 5'-3'
TGF-*β*1	CTTCAATACGTCAGACATTCGGG	GTAACGCCAGGAATTGTTGCTA
Smad-2	ATGTCGTCCATCTTGCCATTC	AACCGTCCTGTTTTCTTTAGCTT
Smad-3	CATTCCATTCCCGAGAACACTAA	GCTGTGGTTCATCTGGTGGT
Smad-7	GACAGCTCAATTCGGACAACA	CAGTGTGGCGGACTTGATGA
*β*-actin	CATCCGTAAAGACCTCTATGCCAAC	ATGGAGCCACCGATCCACA

**Table 2:** Effects on body weight and index of organs.

Groups	Primary body weight (g)	Terminal body weight (g)	Index of liver	Index of spleen	Index of kidneys	Index of testis	Index of thymus
Blank	201.14±9.29	414.71±42.49	2.07±0.13	0.15±0.02	0.60±0.06	0.88±0.10	0.06±0.02
Model	201.00±11.70	418.56±29.29	2.12±0.11	0.14±0.02	0.61±0.05	0.89±0.13	0.04±0.02
Positive	201.44±11.39	425.80±24.06	2.10±0.12	0.16±0.03	0.63±0.06	0.90±0.04	0.05±0.01
Low	201.36±14.14	419.98±10.43	1.98±0.10	0.14±0.04	0.62±0.02	0.89±0.06	0.05±0.01
Medium	198.46±6.91	415.20±33.50	2.12±0.12	0.15±0.02	0.66±0.32	0.86±0.10	0.04±0.01
High	209.11±14.99	430.96±29.42	2.15±0.11	0.13±0.02	0.62±0.04	0.84±0.09	0.05±0.02

Notes: Data are shown as mean±S.E. (n=8 or 9). Terminal body weight means the weight of rats fasted before blood collection.

## Data Availability

All the data related to this article were available from the corresponding author upon reasonable request.
